# Statistical methodology for the analysis of dye-switch microarray experiments

**DOI:** 10.1186/1471-2105-9-98

**Published:** 2008-02-13

**Authors:** Tristan Mary-Huard, Julie Aubert, Nadera Mansouri-Attia, Olivier Sandra, Jean-Jacques Daudin

**Affiliations:** 1 UMR AgroParisTech/INRA 518, 16, rue Claude Bernard 75231 Paris CEDEX 05, France; 2 UMR INRA/ENVA/CNRS 1198, Jouy en Josas, France

## Abstract

**Background:**

In individually dye-balanced microarray designs, each biological sample is hybridized on two different slides, once with *Cy3 *and once with *Cy5*. While this strategy ensures an automatic correction of the gene-specific labelling bias, it also induces dependencies between log-ratio measurements that must be taken into account in the statistical analysis.

**Results:**

We present two original statistical procedures for the statistical analysis of individually balanced designs. These procedures are compared with the usual ML and REML mixed model procedures proposed in most statistical toolboxes, on both simulated and real data.

**Conclusion:**

The UP procedure we propose as an alternative to usual mixed model procedures is more efficient and significantly faster to compute. This result provides some useful guidelines for the analysis of complex designs.

## Background

DNA microarray technology is a high throughput technique by which the expression of the whole genome is studied in a single experiment. Experiments must be well organized and design issues are crucial, see [1,2]. In dual label experiments Cy3 and Cy5 are used as fluorescent dyes allowing to compare two RNA samples on the same slide. It is now well known that there exists a differential effect of the two dyes [3,4], that can be gene-specific. An efficient way to remove this technical artifact is to use balanced reverse dye designs [5]. Balanced reverse dye designs can be divided into three classes along a line of strengthening balancing constraints:

1. Balanced reverse dyes for which each biological sample is hybridized only one time and therefore present with only one dye, on only one array (Table 1.1). These designs are *globally balanced *but not individually balanced.

2. *Individually-balanced *design for which each biological sample is divided into two parts, one hybridized with *Cy*3 on one array and the other with *Cy*5 on another array. Each biological sample is hybridized exactly two times (Table 1.2).

3. Dye-swaps for which each couple of biological samples from two conditions are hybridized on two arrays with reverse dyes. Dye-swaps are constrained to be *couple-balanced *(Table 1.3).

**Table 1:** Three different balanced reverse dye designs for the comparison of 2 treatments

1	array	1	2	3	4	5	6	7	8	9	10
	
	Cy5	A1	B5	A3	B9	A5	B6	A7	B10	A9	B9
	Cy3	B3	A2	B8	A4	B2	A6	B1	A8	B4	A10
2	array	1	2	3	4	5	6	7	8	9	10
	
	Cy5	A1	B1	A2	B2	A3	B3	A4	B4	A5	B5
	Cy3	B1	A2	B2	A3	B3	A4	B4	A5	B5	A1

3	array	1	2	3	4	5	6	7	8	9	10
	
	Cy5	A1	B1	A2	B2	A3	B3	A4	B4	A5	B5
	Cy3	B1	A1	B2	A2	B3	A3	B4	A4	B5	A5

Three different balanced reverse dye designs for the comparison of 2 treatments (A and B), with an equal number of slides. *A*_*i *_stands for the *i*^*th *^biological sample in condition A. (1) Globally balanced design, with 10 biological samples per condition. (2) Individually-balanced design with 5 biological samples per condition. (3) Dye-swap design with 5 biological samples per condition.

Dye-swap design is mostly used when the technical error is higher than the biological variability, either to reduce the technical variance, or when gene-specific dye-bias is of concern [6,7]. When the biological variability is greater than the technical error, *globally balanced *designs are statistically more efficient [5]. However the number of biological samples is sometimes limited, therefore this design is not always possible in practice.

The term *Dye-switch *is used for the first and sometimes also for the second classes. Dye-switch designs of the second class are sometimes described and proposed in papers dealing with microarrays experiments. For example loop designs are often members of this class [8,9], although the distinction between the first and the second class is not always clearly made.

A major point to notice is that the statistical analysis may be very different for the three classes of design. The analysis of the first and third classes is straightforward and well described in articles (see for example [4,10,11]): the experimental units are mutually independent (we consider as usual that the two conjugate arrays of the dye-swaps are summed up to only one experimental unit), and simple statistical procedures such as Student *T*-tests (or regularized *T*-tests) can be performed. On the contrary, if we consider the second class of designs, the experimental units are not independent, a feature that must (or must not) be accounted for. The literature about the statistical study of such designs is limited: some papers proposed some theoretical contributions for their analysis [12,13], but simple guidelines for experimenters and practical considerations (computational burden, choice of a strategy for parameter estimation) are not available.

We consider here the simplest individually-balanced dye-switch design: two conditions *A *and *B *are compared in a two-color cDNA microarray experiment, with *n *biological samples for each condition. The design is the following: each RNA sample (*A*_1 _to *A*_*n *_for condition *A*, and *B*_1 _to *B*_*n *_for condition *B*) is divided into two parts, one labelled with *Cy*5 and the second labelled with *Cy*3. Then 2*n *microarrays are hybridized with respectively *A*_1_*Cy*5 and *B*_1_*Cy*3, *B*_1_*Cy*5 and *A*_2_*Cy*3, *A*_2_*Cy*5 and *B*_2_*Cy*3, and so on till *B*_*n*_*Cy*5 and *A*_1_*Cy*3, (see Table 1.2). There are 2*n *samples, 4*n *labelled samples, 2*n *microarrays, and each sample is hybridized two times (one with *Cy*5 and one with *Cy*3) on two different arrays. We propose a simple, efficient and robust method for the statistical analysis of this experiment.

### Model on the measure of the expression of genes

After the normalization step, *X*_*i *_is the expression measure on the log-scale, for a given gene, corresponding to condition *A *on array *i*. Let *j*(*i*) denote the sample number corresponding to condition *A *and array *i*.

Similarly, *Y*_*i *_is the expression measure for the condition *B *sample on the same array, and *j'*(*i*) the sample number corresponding to condition *B *and array *i*. In the following the gene index is not present in order to simplify the mathematical expressions, but it is important to note that all the terms in the following models are gene-specific. Here we use an analysis of variance (ANOVA) model for the expression measure as introduced by [10].

The model for *X*_*i *_and *Y*_*i *_is the following:

(1) $$\begin{array}{c} X_{i}=\mu_{A}+\delta_{l(i)}+B_{j(i)}+M_{i}+T_{i}\\ Y_{i}=\mu_{B}+\delta_{l^{\prime }(i)}+B_{j^{\prime }(i)}+M_{i}+{T^{\prime }}_{i}, \end{array}$$

where

• *μ*_*A *_and *μ*_*B *_are the population mean expression measures for condition *A *and *B*.

• *δ*_*l*(*i*) _is a two-level fixed effect corresponding to the dye effect. *δ*_*l*(*i*) _= *δ*_1 _(resp. *δ*_2_) for all the samples labelled with Cy5 (resp. Cy3). This term accounts for the *gene-specific dye bias*.

• *B*_*j*(*i*) _represents an independent gaussian random term with mean 0 and standard deviation *σ*_*B*_, corresponding to the random effect of sample *j*(*i*). This variable is specific to the biological sample and is called *biological error*, related to the variability of the biological material inside each population A and B.

• *M*_*i *_represents an independent gaussian random term with mean 0 and standard deviation *σ*_*M*_. *M*_*i *_is the effect of the spot associated to the gene under concern in microarray *i *and has the same value for the two samples which are hybridized on array *i*. This error term takes into account the spatial heterogeneity in each array that affects both *Cy*3 and *Cy*5 measurements.

• *T*_*i *_represents an independent gaussian random term with mean 0 and standard deviation *σ*_*T*_, corresponding to the technical variability, including the steps of labelling, hybridization and measure of intensity of fluorescence. This variable has a specific value for each combination gene×dye×sample, even if the samples are hybridized on the same array and at the same spot, so that *T*_*i *_and ${T^{\prime }}_{i}$ are independent random variables. *T*_*i *_and *M*_*i *_are the two components of the so-called *technical error*.

### Model on the difference of expression on one array

Let *D*_*i *_= *X*_*i *_- *Y*_*i*_*, i *= 1,...,2*n*. Using equation (1) we obtain:

(2) $$D_{i}=\mu_{A}-\mu_{B}+B_{j(i)}-B_{j^{\prime }(i)}+\delta_{l(i)}-\delta_{l^{\prime }(i)}+T_{i}-{T^{\prime }}_{i}$$

which may be written

(3) *D*_*i *_= *μ *+ *BD*_*i *_+ (-1)^*i*+1 ^*δ *+ *TD*_*i*_

where

• *μ *= *μ*_*A *_- *μ*_*B *_is the true differential expression between conditions *A *and *B *for the gene under concern,

• *BD*_*i *_= *B*_*j*(*i*) _-*B*_*j*'(*i*) _is a random variable with mean 0 and standard deviation $\sqrt{2}$*σ*_*B*_,

• *TD*_*i *_= *T*_*i *_- ${T^{\prime }}_{i}$ is an independent random variable with mean 0 and standard deviation $\sqrt{2}$*σ*_*T*_,

• *δ *= *δ*_1 _- *δ*_2 _is the difference between the Cy3 and Cy5 dye effects. This term accounts for the *gene-specific dye bias*.

Each variable *D*_*i *_follows a Gaussian distribution with mean *E*(*D*_*i*_) = *μ *+ (-1)^*i*+1^*δ *and variance $V(D_{i})=2\sigma_{B}^{2}+2\sigma_{T}^{2}$. All the covariances cov(*D*_*i*_, *D*_*j*_) are equal to zero except the following ones:

$$\mathrm{cov}(D_{i},D_{i+1})=\sigma_{B}^{2}$$

with the convention that 2*n *+ 1 = 1.

In this study, we present and compare different strategies for the statistical analysis of individually-balanced designs. The article is organized as follows. In the Results section, five statistical procedures to analyze individually balanced designs (Table 1.2) are compared on both simulated and real data. The Conclusion section draws the main conclusions and gives some useful guidelines for the analysis of individually-balanced designs. The details of the computation are given in the Methods section.

## Results

### Statistical procedure comparison

In this section, we investigate the efficiency of five test procedures for the differential analysis of datasets corresponding to the design of Table 1.2. The procedures are the following (see the Methods section for more details):

• Naive Method NM: for each gene, the naive test statistic

$$T_{N}=\sqrt{2}n\frac{\bar{D}}{\sqrt{S^{2}}}$$

is computed.

• Unbiased Paired Method (UP): for each gene, the unbiased paired statistic

$$T_{UP}=\sqrt{2}n\frac{\bar{D}}{\sqrt{\left(S^{2}+2C\right)}}$$

is computed. Notice that from the Methods section, the theoretical value of *C *must be positive. In practice, the estimated value may be negative. In such a case, *C *is truncated at 0.

• Unbiased Unpaired Method (UU): for each gene, the unbiased unpaired statistic

$$T_{UU}=\sqrt{n}\frac{\bar{X}-\bar{Y}}{\sqrt{S_{X}^{2}+S_{Y}^{2}-2C_{XY}}}$$

is computed. As for the previous method, the value of *C*_*XY *_must be positive. If not, *C*_*XY *_is truncated at 0. Furthermore, the unbiased variance estimator is $S_{X}^{2}+S_{Y}^{2}-2C_{XY}$. Since *C*_*XY *_is non-negative, the variance estimator may have a negative value. In such a case, the variance can be fixed at a given threshold (0.001 in the following).

• Mixed Model with ML estimation (ML): for each gene, model (1) is adjusted with the Maximum Likelihood algorithm.

• Mixed Model with REML estimation (REML): for each gene, model (1) is adjusted with the Restricted Maximum Likelihood algorithm.

It is important to consider both the ML and REML algorithms for the mixed model since each algorithm has its own advantages. While ML is known to provide biased estimates of the variance components, computations are faster and the algorithm does converge. REML gives unbiased estimates of the parameters, but may not converge if the number of observations is small. Both ML and REML computations were performed using the R package Maanova [10].

### Simulations

To study the behavior of the 5 procedures, we performed a simulation study using model (1). We considered 3 different values for $\sigma_{B}^{2}$ (0.5, 1, 2) and $\sigma_{M}^{2}$ (1, 2, 5), 4 values for the number of samples in one condition (5, 10, 20, 30) and 5 possible values for the differential expression *μ *= *μ*_*A *_- *μ*_*B *_(0, 1, 2, 3, 4). The parameter *σ*_*T *_was fixed at 1. For each combination of the parameters, 10,000 genes were simulated.

#### Control of the Type I error rate

We first consider the case *μ *= 0. Table 2 shows the actual Type I error rate level of the 5 test procedures, when the requested nominal level is 5%. Different behaviors can be observed: NM and ML result in a type I error rate higher than the nominal level, and procedure UU is conservative. UP results in an actual level that is close to the expected one, whatever the conditions. In most cases, REML enables an efficient control of the type I error. Yet, when the biological variability is high and the number of samples is low, REML yields a high type I error because of inconsistent estimations of the variance (see the next section). When $\sigma_{B}^{2}$ = 2 and *n *= 5, the discrepancy between the theoretical and the actual level is even worse for REML than for the other methods.

**Table 2:** Actual level of the 5 test procedures in one simulation of 10 000 genes

	$\sigma_{B}^{2}$ = 0.5				$\sigma_{B}^{2}$ = 2			
Method	5	10	20	30	5	10	20	30

Naive	6.9 (0.2)	7.3 (0.2)	7.3 (0.2)	7.5 (0.2)	13.2 (0.3)	13.9 (0.3)	14.0 (0.3)	14.2 (0.3)
Unbiased Paired	5.2 (0.2)	5.2 (0.2)	5.2 (0.2)	5.3 (0.2)	8.2 (0.3)	6.9 (0.2)	6 (0.2)	5.8 (0.2)
Unbiased Unpaired	2.1 (0.1)	1.3 (0.1)	1.0 (0.1)	1 (0.1)	4.6 (0.2)	3.4 (0.2)	2.7 (0.1)	2.9 (0.2)
ML	8.5 (0.3)	8.6 (0.3)	8.3 (0.3)	8.3 (0.3)	12.5 (0.4)	11.1 (0.3)	9.9 (0.3)	9.8 (0.3)
REML	4.7 (0.2)	4.2 (0.2)	4.5 (0.2)	4.9 (0.2)	14.7 (0.4)	8.5 (0.3)	5.9 (0.2)	5.5 (0.2)

Actual mean level (standard error) of the 5 test procedures, for low ($\sigma_{B}^{2}$ = 0.5, left) and high ($\sigma_{B}^{2}$ = 2, right) values of biological variance, and different number of samples *n *in each condition (in column). The requested nominal threshold is 5%.

From these first observations we conclude that we can discard procedures NM and ML, since in differential analysis an effective control of the Type I error rate is necessary.

#### Performance analysis

We now compare the performance of the 3 remaining procedures to detect differentially expressed genes. Table 3 shows the proportion of detected differentially expressed genes, for different values of the parameter set. It clearly appears that the power of procedure UU is low compared with procedures UP and REML. This may be the consequence of the Student approximation (each test statistic is compared with the quantile of a Student distribution with 2*n *- 2 degrees of freedom), that could be more erroneous in the case of the UU statistic.

**Table 3:** Power of the UU, UP and REML test procedures

		*μ *= 1			*μ *= 3		
Nb Samples	$\sigma_{B}^{2}$	UU	UP	REML	UU	UP	REML

5	0.5	5.6	13.6	10.6	55.5	92.1	86.75
5	2	2.8	5.0	12.95	17.9	29.4	34.75
10	0.5	13.2	39.3	33.97	77.8	100.0	99.64
10	2	3.5	7.8	9.06	45.0	63.5	63.06
20	0.5	35.0	80.1	78.13	98.8	100.0	100.0
20	2	7.3	14.5	13.93	82.6	94.8	94.53
30	0.5	51.9	95.5	95.05	100.0	100.0	100.0
30	2	12.1	22.5	21.74	96.2	99.6	99.53

Power (probability of rejecting *H*_0 _× 100) of the different test procedures to detect a low (*μ *= 1, left) or high (*μ *= 3, right) differential expression.

An interesting point is that UP results are more stable than the REML results. If we consider sample sizes *n *larger than 20, we observe that the absolute values of the approximate REML T-test range from 0 to 32, except for some genes where the absolute value is larger than 400. These outliers come from an erroneous estimation of the variance of the mean difference, that is evaluated to be (almost) 0. This does not happen with (UP) since the estimated variance is max(*S*^2^, *S*^2 ^+ 2*C*), i.e. the variance is overestimated to avoid outliers. Notice that despite this overestimation in many cases the power of UP is larger than the power of REML.

#### Computational burden and convergence

We now consider the important question of computational time for the 2 competitive procedures UP and REML. Since microarray experiments can involve hundreds of thousands of genes, it becomes critical to use efficient algorithms for the statistical analysis of the data. Table 4 gives the user CPU time associated to each procedure for the complete analysis of 10,000 genes. While the computational time is constant whatever the condition for the (UP) procedure, (REML) is 8 to 330 times longer than (UP), depending on the number of samples.

**Table 4:** CPU times of procedures UP and REML

*n*	UP CPU	REML CPU	No REML CV
5	2.3	787	56.9
10	2.6	212	5
20	2.8	467	0
30	3.2	1046	0.16

User CPU time of procedures (UP) and (REML), for *σ*^2 ^= 0.5 and different numbers of samples. The last column provides the average number of genes for which REML did not converge.

Furthermore, REML can result in inconsistent estimates of the variance, as shown in the previous sections, or may not converge. Table 4 provides the number of genes for which the REML algorithm did not converge.

### Embriogenomic data

The impact of pregnancy on the cattle endometrium transcriptom is investigated in [14]. In Mammals, the implantation of the embryo is a key event in the establishment of a pregnancy. A microarray experiment has been made to analyze the gene expression of the bovine pregnant endometrium and determine key pathways that control the endometrium physiology during the implantation process. The expression of 13300 genes in the endometrium of cows (n = 5) has been investigated. Only 5 animals were available for each condition so that the dye-switch design of Table 1.2 was used. Gene profiling has been established to analyze the impact of pregnancy by comparing the endometrium of cyclic (day 20 of cycle) versus pregnant animals (day 20 of pregnancy). In the following, the results of the five statistical procedures defined above are compared using this dataset.

**Figure 1:**
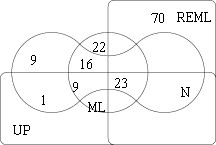
**Venn diagram for the embriogenomics experiment**. Comparison of the DE genes obtained by four methods. Vertical right rectangle: REML, horizontal low rectangle: UP, bone: N and circle: ML.

**Figure 2:**
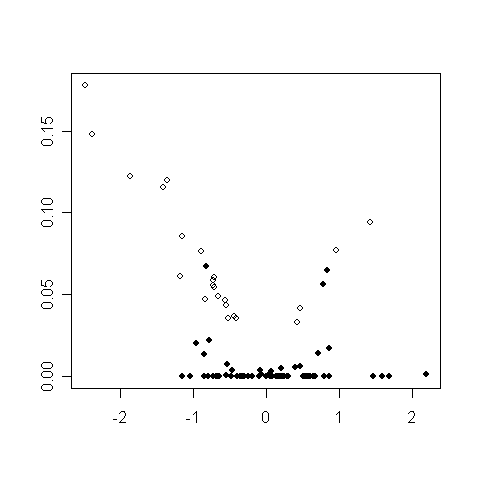
**Comparison of the standard errors obtained with ML, REML and UP for the REML-DE genes of the embriogenomics experiment**. **Left: **REML estimates (y-axis) versus UP estimates (x-axis) of the standard error. **Center: **REML estimates versus ML estimates. **Right: **UP estimates versus ML estimates.

The Venn diagram of Figure 1 shows the number of genes declared differentially expressed (DE) by 4 methods using the Bonferroni method with a 5% level. The UU method gives the least number of DE genes (4) and is not presented in the diagram. REML (which did not converge for 3 genes) gives the greater number of DE genes (93), among which 23 are also found by the other methods, and 70 are specifically found by REML (70 REML specific genes). 70 genes are found DE by ML (22 ML specific genes), and 58 by the naive method (9 Naive specific). Finally 33 genes are declared DE by UP, and all of them are also found by one, two or all of its competitors. Therefore UP provides the less discordant results. The higher number of DE genes obtained with the naive and the ML methods was expected, since it is known from the theory and the simulation study that these methods yield more false positives than the nominal risk. Figure 2 (right) shows that the ML and UP estimates of the standard error are coherent but that the ML estimate are lower than the ones obtained by the UP method. This point is in keeping with the statistical theory which assesses that the UP estimate of the variance is unbiased while the ML estimate has a negative bias.

**Figure 3:**
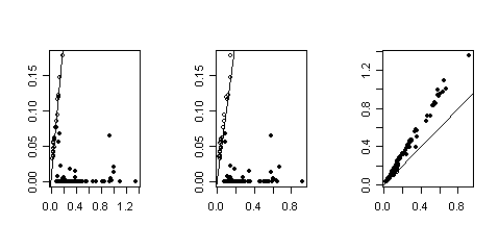
**Mean difference versus standard error for the REML-differentially expressed genes of the embriogenomics experiment**. Standard error of the difference obtained by REML (y-axis) versus mean difference between the two conditions (x-axis). Black points are not found DE by other methods than REML.

The high number of DE genes specifically found with REML is odd. Figures 2 and 3 show that this comes from very low estimates of variance for some genes, so that these genes are declared DE not because the mean difference of expression between the two conditions is high, but because this mean difference is divided by a very low standard error. So most of the 70 genes only found by the REML method are due to too low estimates of the gene variance obtained by the REML algorithm. This observation is in keeping with the results of the simulation study. Therefore the UP method or the naive method should be preferred in this particular experiment. The use of REML without a sharp biological analysis of the results gene by gene would be misleading.

### Teleost fish dataset

**Figure 4:**
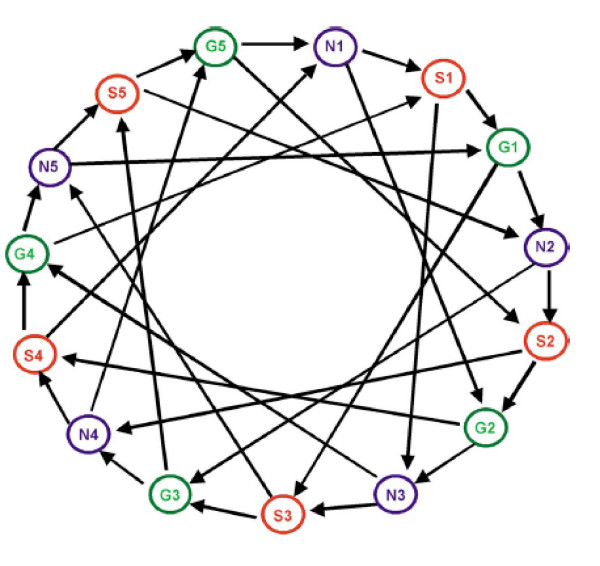
The Teleofish experiment design.

An important application of the methodology proposed in the previous section is the analysis of loop design experiments. Loop and interwoven loop designs were initially proposed in [2] to compare *p *treatments, where *p *is 3 or higher. Figure 4 displays a particular interwoven loop design where 3 different 2-by-2 loop comparisons of treatments are combined in a single experiment. The 3 loop comparisons are

• *N*1 → *S*1 → *N*3 → *S*3 → *N*5 → *S*5 → *N*2 → *S*2 → *N*4 → *S*4 → *N*1

• *S*1 → *G*1 → *S*3 → *G*3 → *S*5 → *G*5 → *S*2 → *G*2 → *S*4 → *G*4 → *S*1

• *N*1 → *G*2 → *N*3 → *G*4 → *N*5 → *G*1 → *N*2 → *G*3 → *N*4 → *G*5 → *N*1

Each of these comparisons corresponds to the design of Table 1.2 discussed in the previous section. Such experimental designs have been studied both theoretically [15] and practically [8,9]. Here, we briefly present the Teleost fish data of [8].

The Teleost fish experiment aims to compare 3 populations of fish (Northern Fundulus heteroclitus, Southern Fundulus heteroclitus and Fundulus grandis). Five individuals were examined in each population to determine the variation in gene expression between populations. Each individual is used to probe four cDNA microarrays, according to the design of Figure 4. The raw data consist of 120 measurements (15 individuals × 4 slides × 2 duplicates per slide) for 907 genes.

In [8], the signal is modelled as follows (after per slide duplicate averaging):

(4) *Y*_*ijkg *_= *m *+ *A*_*i *_+ *D*_*j *_+ (*AD*)_*ij *_+ *G*_*g *_+ (*AG*)_*ig *_+ (*DG*)_*jg *_+ (*V G*)_*kg *_+ *e*_*ijkg*_,

where *A*, *D*, *G *and *V *stand for Array, Dye, Gene and Variety, respectively. Then the 4 measurements corresponding to a given individual are averaged, and an *F *statistic is computed per gene to check whether the variety effect is significant or not.

This strategy roughly amounts to the UU test procedure of section when the number of treatments is higher than 2. The main difference is that in model (4), the model does not include the array random effect which takes into account the dependency between two measures on the same array. According to the results of section, this implies that the variance estimator is biased, leading to a loss of power.

As an alternative, we perform the statistical analysis using the UP procedure. Each pairwise comparison between 2 varieties is made, and a gene is declared differentially expressed if at least 2 of the 3 tests are significant. Each test is performed at the level 0.02, meaning that for a given gene, the nominal level is roughly 0.001 (3 × 0.02^2 ^for 2 of the 3 tests to be significant under *H*_0 _at level 0.02). This is a good compromise between the 0.01 threshold adopted in the original articles with no correction for multiple testing, and the 0.5 × 10^-4 ^(0.05/907) threshold given by a 5% level per test combined with a Bonferronni multiple testing correction. While the drawback of our strategy is to replace one test by three, the advantage is that the variance estimate is unbiased.

Table 5 gives the Oleksiak original list of differentially expressed genes found with the original method and the UP list of genes found with the UP procedure.

**Table 5:** Lists of genes for the Teleofish experiment

Oleksiak list [8]	UP list
RAN GTP binding protein hypo P FLJ20727 ribosomal protein L27 dihydrolipoamide dehydrogenase GTP binding protein	
Steroidogenic acute regulatory protein hypo P FLJ11275 capping protein muscle Z line orla C4 surface glycoprotein HT7 precursor methionine adeno. regulatory Von Willebrand factor succinate dehydrogenase complex KIAA1481 protein protein disulfide isomerase annexin V	Thioredoxin nascent polypeptide associated dnaK type molec. chap. prec. ribosomal protein S16

Lists of genes whose expression was significantly different between populations. The first 5 genes are found differentially expressed by both methods.

**Figure 5:**
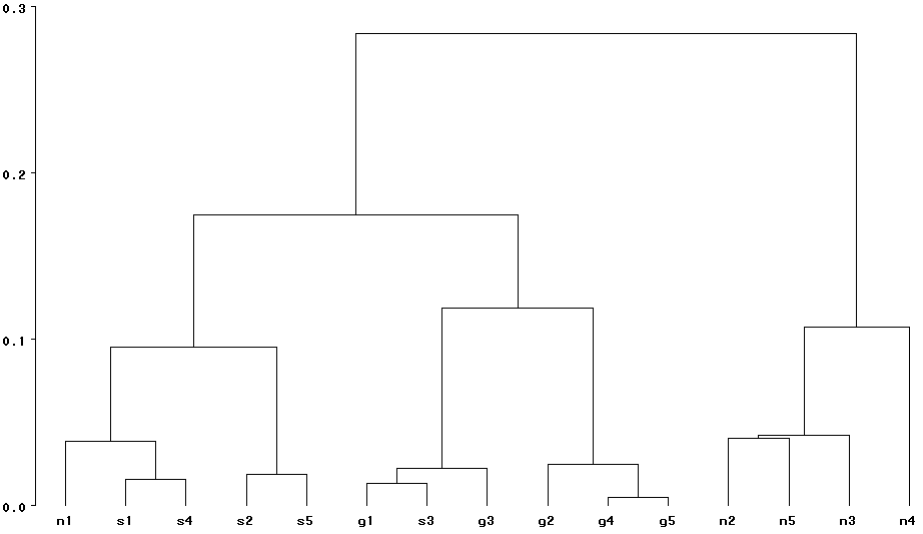
**Clustering tree for the Teleofish dataset**. Clustering tree for the Teleofish dataset, obtained from the second list of differentially expressed genes of Table 5.

Among the 15 genes originally identified, 5 are also declared differentially expressed with the (UP) method. At a first glance, the (UP) procedure seems less powerful than the original method since only 9 genes are found here compared with the 15 genes of the original article. But due to the threshold adopted by the authors in [8], the expected number of false positives is 9 for the Oleksiak list, whereas for the (UP) list we expect only 1 false positive. Therefore most of the 10 extra genes found in [8] may be false positives. To examine the discriminant effect of the 9 genes of the (UP) list, we performed as in the original publication a clustering of the individuals, according to the significative genes. The corresponding tree is given in Figure 5. A cutoff of the tree at 0.15 gives the following 3 classes :

{*S*1, *S*2, *S*4, *S*5, *N*1},   {*G*1, *G*2, *G*3, *G*4, *G*5, *S*3},   {*N*2, *N*5, *N*3, *N*4}.

These 3 classes roughly correspond to the three populations of interest, up to 2 misclassified observations. In the original article, the partition in 3 classes gave

{*N*1, *N*2, *N*3, *N*4, *N*5},   {*S*1, *S*4},   {*G*1, *G*2, *G*3, *G*4, *G*5, *S*2, *S*3, *S*5}.

With only 9 genes (rather than 15), the classification obtained with (UP) is improved compared with the classification of the original method.

## Discussion

Random terms taking into account the array and the sample effects must be included in the statistical model at the gene level for dye-switch experiments. We showed on simulations that the naive paired T-test, which does not take into account the biological sample effect, leads to more false positives than expected, especially when the biological sample effect is high. It may be safely used only when the biological variance is lower than the technical variance. The REML estimate for mixed model provides an approximatively correct false positive rate, at the price of high computational complexity, lack of convergence for low or medium sample sizes and sometimes spurious results. To the contrary, the UP method we propose is easy to implement and not computationally intensive. The method is protected against spurious results, leading to a more robust and powerful analysis than REML when the biological variability is high and the number of samples low, an usual situation in microarray experiments.

For small sample size experiments, it is advised to use regularized T-test, see [16-19]. Regularization strategies are based on statistical methods that take the individual variance of each gene as input and give a regularized variance for each gene as output. The UP procedure proposed in this paper gives an estimate for the variance of the differential expression for each gene, so it allows a further application of all these regularization methods.

## Conclusion

In this paper the proposed estimate of the variance of the differential expression is assessed for the comparison between two conditions in a dye-switch design. The same methodology could be extended to more complex designs involving more than two conditions and duplicate hybridizations of the same biological sample on different arrays.

## Methods

### Paired test procedure

According to expression (2), an unbiased estimator of *μ *is $\bar{D}=\frac{1}{2n}\sum D_{i}$. The variance $V_{\bar{D}}=V(\bar{D}\text{)}$ of this estimator is

(5) $$\begin{array}{lll} V_{\bar{D}} & = & V(\frac{1}{2n}\sum D_{i})\\ & = & \frac{1}{4n^{2}}\left[\sum V(D_{i})+2\sum \mathrm{cov}(D_{i},D_{i+1})\right]\\ & = & \frac{1}{4n^{2}}\left[2n(2\sigma_{B}^{2}+2\sigma_{T}^{2})+4n\sigma_{B}^{2}\right]\\ & = & \frac{1}{2n}(4\sigma_{B}^{2}+2\sigma_{T}^{2})\\ & = & \frac{1}{2n}(V(D)+2\mathrm{cov}(D_{i},D_{i+1})) \end{array}$$

To perform a statistical test on parameter *μ*, we need to estimate $V_{\bar{D}}$.

#### Naive variance estimate

The naive estimate of $V_{\bar{D}}$ is

$$\frac{1}{2n-1}\sum_{i}{\left(D_{i}-\bar{D}\right)}^{2}$$

which is used to perform paired *T*-tests. But in a dye-switch experiment the variables *D*_*i *_- $\bar{D}$ are not centered, since the means of *D*_*i *_and $\bar{D}$ are *μ *+ (-1)^*i*+1 ^*δ *and *μ*, respectively. Hence we consider the alternative estimator

$$S^{2}=\frac{1}{2n-2}\sum_{i}{\left(D_{i}-{\bar{D}}_{\left(i\right)}\right)}^{2},$$

where

$$\begin{array}{ll} {\bar{D}}_{i}=\frac{1}{n}\sum_{i\text{ is odd}}D_{i} & \text{if }i\text{ is odd},\\ {\bar{D}}_{\left(i\right)}=\frac{1}{n}\sum_{i\text{ is even}}D_{i} & \text{otherwise}. \end{array}$$

The expectation of this alternative estimator is

$$\begin{array}{lll} E\left[\frac{1}{2n-2}\sum_{i}{\left(D_{i}-{\bar{D}}_{\left(i\right)}\right)}^{2}\right] & = & \frac{1}{2n-2}\sum_{i}\left[E(D_{i}^{2})-E({\bar{D}}_{\left(i\right)}^{2})\right]\\ & = & \frac{1}{2n-2}\sum_{i}\left[\left(2\sigma_{B}^{2}+2\sigma_{T}^{2})-\frac{1}{n}(2\sigma_{B}^{2}+2\sigma_{T}^{2}\right)\right]\\ & = & \frac{1}{2n-2}\times 2n\times \frac{n-1}{n}(2\sigma_{B}^{2}+2\sigma_{T}^{2})\\ & = & 2\sigma_{B}^{2}+2\sigma_{T}^{2}. \end{array}$$

*S*^2 ^is a downward biased estimator of *V*($\bar{D}$). The higher $\sigma_{B}^{2}$ compared with $\sigma_{T}^{2}$, the higher the bias:

$$V(\bar{D})=E(S^{2})\left[1+\frac{\sigma_{B}^{2}/\sigma_{T}^{2}}{1+\sigma_{B}^{2}/\sigma_{T}^{2}}\right].$$

From this naive estimate of the variance we can derive a first T-test statistic to be used for the differential analysis:

(6) $$T_{N}=\sqrt{2n}\frac{\bar{D}}{\sqrt{S^{2}}}$$

#### Unbiased variance estimate

Let $C=\frac{1}{2n-4}\sum_{i}\left(D_{i}-{\bar{D}}_{\left(i\right)})(D_{i+1}-{\bar{D}}_{\left(i+1\right)}\right)$. We have

$$\begin{array}{lll} E(C) & = & \frac{1}{2n-4}\sum_{i}E[D_{i}D_{i+1}-D_{i}{\bar{D}}_{\left(i+1\right)}-D_{i+1}{\bar{D}}_{\left(i\right)}+{\bar{D}}_{\left(i\right)}{\bar{D}}_{\left(i+1\right)}]\\ & = & \frac{1}{2n-4}\sum_{i}\left[\sigma_{B}^{2}-\frac{2}{n}\sigma_{B}^{2}-\frac{2}{n}\sigma_{B}^{2}+\frac{1}{n^{2}}\times n\times 2\sigma_{B}^{2}\right]\\ & = & \sigma_{B}^{2}. \end{array}$$

From this and equation (5) we can deduce the following unbiased estimate of $V_{\bar{D}}$:

$$S_{\bar{D}}^{2}=\frac{1}{2n}(S^{2}+2C)$$

Finally, the "unbiased paired t-statistic" for testing the null hypothesis *H*_0 _= {*μ*_1 _= *μ*_2_} is

$$T_{UP}=\sqrt{2n}\frac{\bar{D}}{\sqrt{\left(S^{2}+2C\right)}}$$

which is approximately distributed as a Student distribution with 2*n *- 2 *df *under *H*_0_.

### Unpaired test procedure

Let ${\bar{X}}_{j}$ (respectively ${\bar{Y}}_{j}$) be the mean of the 2 results obtained with the same biological sample (in 2 different arrays and with the 2 dyes) for condition A (respectively condition B). From model (1) one obtains

$$\begin{array}{l} {\bar{X}}_{j}=\mu_{A}+(\delta_{1}+\delta_{2})/2+B_{j}+M_{i(j)}+M_{i^{\prime }(j)})/2+(T_{i(j)}+T_{i^{\prime }(j)})/2\\ {\bar{Y}}_{j}=\mu_{B}+(\delta_{1}+\delta_{2})/2+{B^{\prime }}_{j}+M_{i(j)}+M_{i^{\prime }(j)})/2+({T^{\prime }}_{i(j)}+{T^{\prime }}_{i^{\prime }(j)})/2 \end{array}$$

where *j *is the biological sample index (recall that sample *j *is different for the two conditions), *i*(*j*) and *i'*(*j*) are the arrays on which sample *j *has been hybridized. ${\bar{X}}_{j}$ and ${{\bar{Y}}^{\prime }}_{j}$ may be correlated as a result of a possible common array effect. ${\bar{X}}_{j}$ and ${{\bar{X}}^{\prime }}_{j}$ are uncorrelated because the two different biological samples of the same condition cannot be present together on the same array. From result (5) we have:

$$V(\bar{X}-\bar{Y})=V_{\bar{D}}=\frac{1}{2n}(4\sigma_{B}^{2}+2\sigma_{T}^{2}).$$

The usual unpaired estimate of $V(\bar{X}-\bar{Y})$ is equal to $(S_{X}^{2}+S_{Y}^{2})/n$, where $S_{X}^{2}=\frac{1}{n-1}\sum_{j}{\left({\bar{X}}_{j}-\bar{X}\right)}^{2}$ and $S_{Y}^{2}=\frac{1}{n-1}\sum_{j}{\left({\bar{Y}}_{j}-\bar{Y}\right)}^{2}$, whose common mean (under the homoscedastic model (1)) is equal to

$$\sigma_{B}^{2}+\frac{1}{2}(\sigma_{T}^{2}+\sigma_{M}^{2}).$$

Therefore

$$E\left[\left(S_{X}^{2}+S_{Y}^{2}\right)\right]=2\sigma_{B}^{2}+\sigma_{T}^{2}+\sigma_{M}^{2}.$$

This method overestimates the true variance

$$V_{\bar{D}}=\frac{1}{2n}\left[4\sigma_{B}^{2}+2\sigma_{T}^{2}\right].$$

The overestimation is more dramatic as $\sigma_{M}^{2}$ increases. This estimate may be corrected: $\sigma_{M}^{2}$ may be estimated using the empirical covariance between ${\bar{X}}_{j}$ and ${\bar{Y}}_{j^{\prime }}$. Let

$$C_{XY}=\frac{1}{n-2}\left(\sum_{j=1}^{n}\left({\bar{X}}_{j}-\bar{X})({\bar{Y}}_{j}-\bar{Y}\right)+\sum_{j=1}^{n}\left({\bar{X}}_{j}-\bar{X})({\bar{Y}}_{j-1}-\bar{Y}\right)\right)$$

with the convention that ${\bar{Y}}_{0}={\bar{Y}}_{n}$. The mean of the first sum is

$$\begin{array}{lll} \frac{1}{n-2}\sum_{j=1}^{n}E\left[\left({\bar{X}}_{j}-\bar{X})({{\bar{Y}}^{\prime }}_{j}-\bar{Y}\right)\right] & = & \frac{1}{n-2}\sum_{j=1}^{n}E\left[\left((M_{i(j)}+M_{i^{\prime }(j)})/2-\bar{M})((M_{i(j)}+M_{i^{\prime \prime }(j)})/2-\bar{M}\right)\right]\\ & = & \frac{1}{n-2}\sum_{j=1}^{n}\frac{\sigma_{M}^{2}}{4}-\frac{2\sigma_{M}^{2}}{4n}\\ & = & \frac{\sigma_{M}^{2}}{4} \end{array}$$

It is easy to see that the second sum in *C*_*XY *_has the same mean. Therefore an unbiased estimate of $V_{\bar{D}}$ is $\frac{1}{n}(S_{X}^{2}+S_{Y}^{2}-2C_{XY})$, and the approximate unpaired t-statistic is

(7) $$T_{UU}=\sqrt{n}\frac{\bar{X}-\bar{Y}}{\sqrt{S_{X}^{2}+S_{Y}^{2}-2C_{XY}}}.$$

## Authors' contributions

TMH and JJD conceived the method and prepared the manuscript. TMH and JA implemented part of the software and performed the statistical analysis. NM made the Embriogenomics experiment under the direction of OS. All authors contributed to the discussion and approved the final manuscript.
